# From AIBO to robosphere. Organizational interdependencies in sustainable robotics

**DOI:** 10.3389/frobt.2025.1716801

**Published:** 2025-12-18

**Authors:** Antonio Fleres, Luisa Damiano

**Affiliations:** Department of Communication, Art and Media, IULM University, Milan, Italy

**Keywords:** AIBO, robosphere, self-organization, social robotics, socio-technical ecosystems, sustainability

## Abstract

The challenge of sustainability in robotics is usually addressed in terms of materials, energy, and efficiency. Yet the long-term viability of robotic systems also depends on organizational interdependencies that shape how they are maintained, experienced, and integrated into human environments. The present article develops this systemic perspective by advancing the hypothesis that such interdependencies can be understood as self-organizing dynamics. To examine this hypothesis, we analyze the case of Sony’s AIBO robotic dogs. Originally designed for social companionship, AIBO units gave rise to a hybrid socio-technical ecosystem in which owners, repair specialists, and ritual practices sustained the robots long after their commercial discontinuation. Building on self-organization theory, we introduce the concept of the “robosphere” as an evolving network of relations in which robotic and human agents co-constitute resilient, sustainability-oriented ecosystems. Extending self-organization beyond its classical biological and technical domains, we argue that robotic sustainability must be framed not as a narrow technical issue but as a complex, multifactorial, and distributed process grounded in organizational interdependencies that integrate technical, cognitive, social, and affective dimensions of human life. Our contribution is twofold. First, we propose a modeling perspective that interprets sustainability in robotics as an emergent property of these interdependencies, exemplified by repair, reuse, and ritual practices that prolonged AIBO’s lifecycle. Second, we outline a set of systemic design principles to inform the development of future human–robot ecosystems. By situating the AIBO case within the robospheric framework, this Hypothesis and Theory article advances the view that hybrid socio-technical collectives can generate sustainability from within. It outlines a programmatic horizon for rethinking social robotics not as disposable products, but as integral nodes of co-evolving, sustainable human–robot ecologies.

## 1 Introduction: human–robot ecologies and the challenge of sustainability

The increasing diffusion of robotics across therapeutic, industrial, artistic, military, urban, and domestic domains is reshaping not only technological infrastructures but also human social environments. Developments in multi-agent systems illustrate a tendency to connect artificial agents in decentered and distributed ways, reinforcing the view of robotics as a technically networked domain—a perspective captured by the notion of “networkism” (Novikov, 2015). Yet the rise of service and social robots shows that artificial agents are not confined to technical interconnections: through their interactive and relational capacities, they increasingly participate in social networks alongside human agents. In this sense, hybrid human–robot ecologies emerge as sites where technological, human, and social systems become entangled, extending networked logics into genuinely socio-technical domains.

These hybrid ecologies also raise pressing questions of sustainability, as their long-term viability depends not only on material efficiency but also on their organizational resilience and social relevance.

Building on this background, recent theoretical work has used the term “robosphere” - originally introduced by Maturana and Bunnell (1998) - to describe a newly emerging complex system composed of interrelated human and robotic agents (e.g., Colombano, 2003; Dudley-Rowley and Colombano, 2004; Lamola, 2022; Fleres, 2025). Different philosophical perspectives have engaged with this notion, from post-phenomenological interpretations focused on meaning-making (Lamola, 2022) to systemic interpretations grounded in complex systems theory (Fleres, 2025), which emphasize the dynamics of interaction, closure, and homeostasis. In this paper, we adopt the latter perspective, viewing the robosphere as an evolving network of self-organizing hybrid ecosystems that can sustain themselves over time. Specifically, we take the case of AIBO, SONY’s iconic robotic pet, to illustrate how such systems can sustain their coherence and continuity through spontaneous coordination and organizational dynamics.

Our original contribution is twofold: first, we advance a sustainability-oriented reinterpretation of the robosphere, grounded in self-organization theory and extending autonomous system modeling beyond its classical domains to hybrid human–robot ecologies; second, we outline preliminary design principles for resilient, socially relevant human–robot ecosystems, illustrated through the AIBO case.

This paper is organized as follows. Section 2 reviews related work, situating our contribution within the broader field of sustainable robotics and socio-technical systems. Section 3 formulates the research question and outlines the methodology adopted in this study. Section 4 presents the AIBO case, which serves as the empirical basis for our analysis. Section 5 introduces the theoretical model of a generic self-organized system, providing the conceptual framework for interpretation. Section 6 offers a detailed account of how the AIBO case aligns with and exemplifies the model described in the preceding section. Section 7 discusses the sustainability implications of the AIBO case, highlighting how a self-organizational perspective can shed light on sustainable practices and dynamics. Section 8 proposes a preliminary set of principles for the development of sustainable socio-technical ecologies, derived from the insights of the case study. Finally, Section 9 concludes the paper and outlines directions for future research.

## 2 Background: AIBO, HRI, and sustainable robotics

This section reviews prior research across two different domains: studies on AIBO and human–robot interaction (HRI), sustainability in robotics. While each of these areas has evolved independently, their intersection remains underexplored. This paper seeks to bridge this gap by reinterpreting the case of AIBO through self-organization theory, thereby advancing a novel theoretical approach to understanding sustainable socio-technical systems in robotics.

### 2.1 Studies on AIBO: Emotional engagement, attachment, and long-term use

The Sony AIBO robot dog has garnered attention not only for its innovative technological features but also for its potential impact on emotional and psychological wellbeing. Various studies have explored the emotional responses and social interactions humans exhibit towards AIBO, indicating that such robotic companions could serve as significant tools in therapy and companionship.

Much of the research on AIBO has focused on the emotional and affective dynamics of human–robot attachment, highlighting how relational experiences with this robot go far beyond its technical affordances.

One notable aspect of research on AIBO is the exploration of human-robot attachment. Studies conducted by Dobai et al. illustrated the development of a personality model for AIBO that correlates users’ emotional responses with interaction patterns, suggesting that AIBO can positively influence users’ moods (Dobai et al., 2005). Similarly, Komatsu and Yokoyama examined the psychological dynamics in human-robot interactions, revealing that intimate relationships could be fostered with AIBO despite the inherent challenges (Komatsu and Yokoyama, 2007). Expanding on this, Lee et al. investigated how the perceived developmental capabilities of AIBO influenced users’ feelings of social presence and attachment over a month-long interaction period, highlighting that participants responded more positively to robots perceived as developing rather than fully matured (Lee et al., 2005).

Moreover, AIBO has demonstrated potential in therapeutic settings. Studies indicate that interactions with AIBO can elicit positive emotional outcomes, aiding in mental health therapy, particularly for individuals suffering from loneliness or stress. This is echoed in findings that demonstrate the impact of robotic companions on reducing emotional challenges among older adults (Alhouli et al., 2023). Krueger et al. discussed the similarities in attachment forms between humans and robots, suggesting that understanding these relationships through the lens of companion animals can enhance our comprehension of human-robot interactions (Krueger et al., 2021).

Another significant area of exploration is the long-term acceptance and relationship dynamics between users and AIBO. Kertész and Turunen provided insight into the experiences of long-term users, revealing the evolving expectations and emotional attachments that develop over years (Kertész and Turunen, 2017). Their investigation into heavy AIBO users highlighted how prolonged interactions can facilitate deeper emotional bonds, paralleling those found in traditional human–animal relationships (Díaz-Boladeras, 2023). Additionally, Wong et al. indicated that users often attribute personality traits to AIBO, enriching the interaction experience and shaping users’ emotional engagements with the robot (Woods et al., 2007).

The design and functionality of AIBO have also been scrutinized regarding emotional engagement. Reviews of affect detection methodologies (McColl et al., 2016) and studies of adaptive behavior (Pepe et al., 2007) emphasize how social robots like AIBO can engage in more naturalistic interactions, fostering empathetic connections.

The research on AIBO spans psychology, medicine, and robotics, highlighting the multifaceted impacts of robotic companions. Both the emotional attachment formed, and the therapeutic potentials exhibited by AIBO underscore the importance of continued exploration into human-robot interactions to better understand and leverage these relationships in practical and clinical contexts.

Taken together, these findings show that AIBO is not only a site of technological and therapeutic innovation but also a generator of social and affective practices that sustain its presence over time. This tendency toward preservation, attachment, and repair suggests a direct link between affective HRI and the broader challenge of sustainability in robotics.

### 2.2 Sustainability in robotics: from materials to systems

The push for sustainability within the robotics sector is gaining momentum as researchers and engineers seek to minimize the environmental impacts associated with robotic technologies.

So far, most approaches have concentrated on material and technical innovations, while the social and organizational dimensions of sustainability remain less explored.

One prominent strategy is the use of biodegradable materials in robotic construction. Yuki emphasizes that incorporating biodegradable components can significantly mitigate environmental impacts, particularly in situations where robots may become lost or discarded in natural settings Yuki (2019). This aligns with Murakami et al., who discuss pioneering developments in “plant robots” that employ natural materials which grow and decompose, raising the potential for eco-friendly robotic systems that harmonize with their surroundings while demonstrating the principles of sustainability (Murakami et al., 2024). These studies underscore the growing need for eco-friendly materials in robotics, a sentiment echoed by Nagai et al., who analyze various biodegradable actuators to design effective sustainable robotics (Nagai et al., 2021).

Additionally, innovations in soft robotics represent a critical advancement towards sustainability. Rumley et al. focus on the development of biodegradable electrohydraulic actuators, which are essential for soft robots expected to perform tasks in sensitive environments (Rumley et al., 2023). Such technologies not only reduce reliance on conventional materials but also facilitate the design of robots that can decompose without leaving harmful residues. This line of research is reinforced by Wei et al., who introduce strategies for creating fully biodegradable origami-based robots, emphasizing a closed-loop sustainability approach, where robots can disintegrate without detrimental environmental effects (Wei et al., 2025). This holistic view extends to the lifecycle of robotic systems, ensuring that as technologies evolve, their material components do not contribute to long-term pollution.

Moreover, the integration of machine learning and artificial intelligence into robotics can also drive sustainability efforts. Chen et al. discuss how enhanced robotic perception, and decision-making capabilities can facilitate more efficient operations, ultimately aligning with several United Nations Sustainable Development Goals, including responsible consumption (Chen et al., 2024). These advancements enable robots to operate more efficiently, thus reducing energy consumption and waste during manufacturing processes.

On a broader industrial scale, the implications of robotic deployment on urban carbon emissions highlight their potential as tools for sustainability. Zhang discusses how integrating industrial robots can lead to reduced carbon footprints through enhanced productivity and the promotion of green technologies within urban environments (Zhang et al., 2025). Such findings suggest that robots need not only to be designed for immediate efficiency but also must be envisioned as long-term contributors to sustainable urban development.

Furthermore, soft robotics continues to show promise within the context of sustainable practices. Giordano et al. highlight how improvements in understanding biological principles and utilizing eco-friendly materials are crucial to developing robots that are inherently suited for environmental sustainability (Giordano et al., 2023). They argue for the need to explore renewable energy sources as part of soft robot design, preparing the way for more autonomous and sustainable solutions.

Building on developments in soft robotics, an emerging area in sustainable robotics is necrobotics, which explores the use of biotic material as functional components in robotic systems. Yap et al. have shown that biotic structures can serve as actuators, offering a pathway toward biodegradable alternatives to conventional synthetic parts (Yap et al., 2022). This approach has the potential to reduce reliance on non-renewable resources and limit the accumulation of technological waste. While still in its experimental stage, necrobotics suggests novel strategies for robotic fabrication that align more closely with ecological cycles of use, decay, and regeneration.

Finally, the field also benefits from multi-disciplinary approaches that combine various technologies. Hartmann et al. emphasize the necessity of integrating sustainable energy management with soft robotics technologies to address rising energy demands and environmental concerns (Hartmann et al., 2021). This convergence of disciplines is vital for developing systems that perform tasks efficiently while operating within an environmentally conscious framework.

The expansion of sustainable practices in robotics is marked by innovative materials, improved operational methodologies, and the integration of artificial intelligence. By fostering a generation of robots designed with sustainability at their core, researchers are laying the groundwork for technologies that fulfill functional needs while being environmentally responsible.

Yet, while crucial, these strategies focus almost exclusively on materials and energy. What remains largely unaddressed are the social, affective, and organizational dynamics through which human–robot ecosystems — such as the AIBO community — generate sustainable practices over time. Recognizing that material solutions are necessary but not sufficient, in this paper we introduce a complementary approach grounded in self-organization theory and the notion of the robosphere, whose conceptual background is outlined below.

#### 2.2.1 Self-organizing approaches in contemporary robotics

Since the development of the embodied approach in AI (e.g., Brooks, 1991; Pfeifer and Scheier, 1999), robotics has progressively moved away from centralized control architectures and toward models grounded in distributed coordination, environmental coupling, and behavior emerging from local interactions. Building on this shift, the early 2000s saw the consolidation of a coherent field of *self-organizing robotics*, encompassing research areas such as swarm robotics, soft robotics, bio-inspired robotics, and circular robotics (e.g., Pfeifer et al., 2007). Despite their methodological diversity, these approaches share an interest in how coherent behavioral patterns arise from decentralized interactions among heterogeneous components.

These directions resonate with classical work in complex systems (e.g., Weiss, 1974; Prigogine and Stengers, 1979; Varela, 1979; Jantsch, 1980; Kauffman, 1995; Capra, 1996; Solé and Goodwin, 2000; Solé and Bascompte, 2012), which describe biological, ecological, social, and technical systems as governed by self-organizational and dynamical principles of emergence, adaptation, and coordination. Within this broader lineage, self-organization theory provides a conceptual framework for interpreting robotic systems not only as engineered artefacts but as participants in multi-level ecologies of interaction.

This perspective appears particularly relevant for social and companion robots such as AIBO, whose long-term evolutionary trajectory emerges from the interplay of technical, affective, and material dynamics within their user communities.

Seen from this angle, the notion of the *robosphere*, as developed in the next subsection, emerges as a useful conceptual tool for characterizing socio-technical environments in which human and robotic agents jointly contribute to evolving organizational patterns. This conceptual positioning, while clarifying the relevance of the robosphere framework within the broader background of this study, prepares the ground for the methodological approach and model we adopt.

### 2.3 Robosphere as a framework for sustainable robotics

In line with this perspective, in this study we adopt and further develop the notion of the robosphere proposed in Fleres (2025), which explicitly establishes an additional bridge between robotics and self-organization theory. When situated within self-organizational and complex systems approaches, the concept of the robosphere emerges as a valuable analytical framework for addressing sustainability in robotics. This is because the challenge of sustainability is inherently tied to complexity—it arises from a failure to adequately grasp and respond to the dynamics of complex systems, often due to reductionist approaches. Reconceptualizing the robosphere within complexity theory offers a shift in perspective: it situates the robot within a factual reality that is neither simple nor linear. The robosphere invites us to abandon reductionist perspectives—such as the binary logic often embedded in Human-Robot Interaction (HRI)—in favor of a systemic vision. The definition of the robosphere as it was proposed by Fleres (2025) frames it as the totality of integrative human–robot ecologies, or socio-technical systems, governed by the principles and dynamics typical of complex systems. Within these systems, robots are not isolated entities but function as nodes within broader networks, capable of interacting with other elements.

This standpoint allows us to integrate the two strands of research reviewed above — on AIBO/HRI and on sustainable robotics — into a unified perspective. It highlights how sustainability in robotics is not only a matter of material efficiency but also emerges from affective bonds, repair cultures, and collective practices that extend the lifecycles of robotic agents.

The robospheric perspective highlights the role of artificial empathy in sustaining such dynamics, including long-term engagement, repair culture, and resource circularity. By applying the generic model of self-organizing systems to the AIBO ecosystem, this work shows how sustainability can be understood as an emergent property of hybrid human–robot collectives.

In this way, the article situates itself at the intersection of research on AIBO and HRI and the growing field of sustainability in robotics, grounding its analysis in the systemic perspective of the robosphere. The contribution advanced here opens a path for exploring sustainability in robotics that complements established approaches focused on material innovation and energy efficiency. Rather than concentrating exclusively on technological components and their optimization, it foregrounds the relational dynamics of socio-technical systems, showing how sustainability emerges from organizational and affective interdependencies that shape human–robot ecologies.

## 3 Methodology and research questions

Building on the conceptual background developed in the previous sections, this study investigates the socio-technical ecosystem that formed around Sony’s AIBO robot by adopting a conceptual and theoretical approach. The aim is to examine whether the organizational dynamics observed in this hybrid community can be coherently interpreted through the notion of the robosphere and the self-organizational framework introduced earlier. The following subsections present the methodological grounding of the study, explain how the AIBO case is situated within this framework, introduce the questions that guide the analysis, and describe the general steps through which the case is examined.

### 3.1 Epistemological case analysis

This methodological approach is consistent with a long-standing tradition in complex systems research, where specific cases are analyzed to evaluate and express the explanatory power of general models of self-organization. Authors such as Francisco Varela (1979), Eric Jantsch (1980), Ilya Prigogine (1981) and Kauffman (1995) have used concrete systems—ecological, organizational, and social—to examine how self-organizing principles manifest across heterogeneous domains.

Varela’s work offers one of the most rigorous formulations of this tradition. In *Principles of Biological Autonomy* (1979) and in several later writings, he explicitly treats autonomy and closure as cross-domain organizational patterns that can be identified in biological, technical, and social systems. In this framework, organizational closure functions as an epistemic criterion for assessing whether a given system instantiates autonomous organization (Damiano, 2009; Di Paolo and Thompson, 2025).

By following this methodological lineage, the present study uses the AIBO community not as an object of sociological observation, but as an epistemological case through which to evaluate whether general self-organizational features—as defined in the generic model of self-organizing systems adopted here based on Varela’s work (see Section 5)—can be meaningfully identified in a hybrid human–robot ecology. The analysis is guided by conceptual criteria drawn from this tradition, focusing on how distributed interactions contribute to system-level coherence; whether global patterns arise that are not reducible to individual components; the extent to which organizational closure and autonomous dynamics can be discerned; and how technical, relational, and material dimensions co-evolve over time. Together, these elements define the conceptual grounding from which the AIBO case is examined. In order to ensure analytical consistency, the operational framework adopted in this study is the generic model of autonomous self-organizing systems derived from the Varelian tradition. For clarity of exposition, this model is presented in Section 5 before being applied to the AIBO case in Section 6.

### 3.2 Case selection and data sources

Among existing examples of long-term human–robot interactions, the case of AIBO offers particularly facilitating characteristics for the assessment of the explanatory potential of self-organization theory. Its historical and relational trajectory—introduced in general terms in Section 2 and analyzed empirically in Section 4 of this paper—provides conditions that are especially suitable for evaluating whether the organizational descriptors associated with self-organization can illuminate the dynamics of a hybrid socio-technical ecology.

As empirical basis this study uses a heterogeneous set of publicly available sources, including ethnographic accounts, historical reconstructions, journalistic documentation, and technical descriptions of maintenance and repair infrastructures. Rather than treating these materials as datasets to be coded, they were treated in the analysis as diverse forms of evidence for evaluating whether the organizational descriptors, derived from self-organization theory, supply conceptual coherence when applied to a real-world human–robot system. This use of heterogeneous secondary documentation is consistent with the epistemological case methodology and with the broader tradition outlined above.

### 3.3 Research questions

The study is guided by two central research questions:

1. Can the AIBO hybrid community be meaningfully interpreted as part of the robosphere when analyzed through self-organization theory, and what does this reveal about the sustainability of socio-technical ecosystems?
2. Which dynamics identified in this case can inform preliminary design principles for more sustainable and resilient robotic systems?

### 3.4 Procedural steps

The analysis proceeds in the following three steps.

- Case study analysis: a detailed reconstruction of AIBO’s lifecycle, focusing on its discontinuation and the subsequent emergence of community-driven repair and reuse practices.
- Conceptual application: an interpretation of the AIBO case through the descriptors of the generic model of self-organizing systems, identifying organizational characteristics such as organization, emergence, autonomy, co-evolution, and closure.
- Theoretical elaboration: an exploration of how this interpretation of AIBO as a self-organizing system can generate insights into the sustainability potential of human–robot ecosystems, expanding the robosphere framework into a sustainability-oriented perspective.

This methodological choice reflects an integrative understanding of sustainability that encompasses social, environmental, and economic dimensions, consistent with the orientation of the Research Topic. By examining how user-driven repair, reuse, and stewardship practices sustained AIBO after corporate withdrawal, the study highlights how resilience and circularity can emerge from affective and organizational interdependencies. Building on this analysis, we propose a preliminary set of design principles intended to guide the next-generation of robotics toward a more socially aware and sustainability-oriented development of hybrid human–robot ecosystems.

## 4 The case of AIBO: from discontinuation to hybrid community

### 4.1 AIBO: a robot profile

AIBO is a robotic dog, designed to function as a companion, which was developed, manufactured by SONY between 1999 and 2006. More than 150,000 units were sold worldwide during this period. The name “AIBO” is an acronym for “Autonomous Intelligent Robot,” and it resembles the Japanese word *aibō*, meaning “friend” or “companion”, in line with its intended role as a robotic pet destined to social interaction with its owners (Kahn et al., 2002). AIBO is a robotic companion equipped with a range of sensors—including tactile and environmental sensors—that enable both verbal and physical interaction with users.

It can express up to six distinct emotions through its embodied behaviour, allowing it to engage with humans in ways reminiscent of a domestic pet (Pfeifer and Bongard, 2006; Sharkey and Sharkey, 2012). These features support AIBO’s use in various contexts, notably as a social companion, and in therapeutic settings, particularly among elderly individuals and children (Weiss et al., 2009; Kanamori et al., 2002; Kertész and Turunen, 2019). Some AIBO models also support the installation of a software package called *AIBOLife*, which allows the robot to develop a unique personality over time, shaped by its interactions with the user.

### 4.2 Post-production stewardship: repair, recycling and rituals

In January 2006, SONY announced its decision to discontinue the production of AIBO. Support for the robot was progressively reduced, culminating in its complete termination in 2013. In 2014, SONY formally ended all official replacement and repair services, leaving users without authorized options for maintenance. In response, former SONY engineer Norimatsu Nobuyuki founded *A-Fun* in 2011, a company explicitly aimed at meeting the specific needs of owners whose emotional attachment to AIBO was profound (Burch, 2018).

A-Fun addressed the shortage of spare parts by salvaging components from donated AIBO units. Each robot underwent a repairability assessment; those deemed beyond repair were formally declared “dead” and dismantled to provide parts for others. To acknowledge owners’ grief, A-Fun partnered with Buddhist monk Ōi Bungen, who officiated memorial services for donated AIBOs (White and Katsuno, 2021). Between 2015 and 2018, approximately 700 mortuary rites were held. These practices, combining repair, reuse, and ritual, did more than maintain individual units: they extended AIBO’s operational lifespan and generated forms of resource circularity with clear sustainability implications (Knox and Watanabe, 2018).

### 4.3 End-of-life rituals and resource circularity

The end-of-life phase of AIBO reveals how technical and emotional dimensions intertwine to sustain a hybrid ecology. Mortuary ceremonies transformed the dismantling of obsolete robots into a socially meaningful act, recognizing the grief of owners and affirming their bond with the robot community. Studies show that human responses to the “death” of robots can mirror those to the loss of pets or even humans, highlighting the depth of personification and affective engagement (Carter et al., 2020). At the same time, the donation of components from “dead” units ensured a circular economy model, where obsolete devices became valuable resources for sustaining others. These practices exemplify how affective bonds and organizational arrangements jointly support sustainability, ensuring that robotic companions remained active long after corporate withdrawal.

### 4.4 AIBO hybrid community as a research object

This virtuous dynamic of life-cycle extension can be understood through two key factors.

The first concerns the emotional bonds that social robots like AIBO create with their users. Literature in social robotics highlights that the effectiveness of these robots—often described as *social presence* (Biocca et al., 2003; Dumouchel and Damiano, 2017)—is grounded in affective signals. This is frequently framed as *artificial empathy*, a systemic form of affective coordination established through mutual interaction (Damiano and Dumouchel, 2020). In AIBO’s case, this ability was decisive: its adaptive personality and emotional responsiveness enabled co-evolutionary relationships that motivated owners to repair rather than replace, extending the robot’s lifespan (Melson et al., 2009).

The second factor is the rise of specialized repair companies, particularly A-Fun, which ensured technical support after SONY’s withdrawal. This organizational actor sustained the continuity of the ecosystem, enabling users to maintain their robots against obsolescence.

Unlike ordinary electronic devices, AIBO was designed for social interaction, which explains why its discontinuation led to ritual practices and sustained communities rather than simple disposal. This hybrid community—integrating owners, robots, and repair services—thus became a genuine research object for examining the interplay between affect, organization, and sustainability.

### 4.5 Theoretical framing of the AIBO hybrid community

The hypothesis guiding this study is that the interdependent network formed by owners, robots, and A-Fun can be described as a self-organized system that is both resilient and sustainability-oriented. It resists dominant models of rapid consumption by promoting reuse, repair, and resource valorization. More than an example of affective human–robot interaction, the AIBO case exemplifies the emergent autonomy of heterogeneous collectives, integrating emotional, organizational, and technical elements.

This raises the question of how such a hybrid system can be meaningfully framed as self-organizing and situated within broader theories of sustainability. To address this, the following section introduces the generic model of self-organizing systems (Varela, 1979; Damiano, 2009; 2012; Fleres, 2025), which provides the conceptual framework for our analysis. In this way, the AIBO community shows how sustainability can emerge not only from technical reuse but also from organizational and affective interdependencies — an exemplary instance of the robosphere as a sustainability-oriented human–robot ecosystem.

## 5 Theoretical reference model: the autonomous or self-organized system

The theoretical reference model guiding our interpretation of the AIBO system is the generic model of self-organizing systems presented in Damiano (2009), Damiano (2012). This model was constructed by identifying conceptual convergences among pioneering approaches to self-organization. Here, *generic* indicates that the model is independent of specific levels or domains of application, while *pioneering approaches* refers to the foundational scientific frameworks that first introduced the notion of self-organization into scientific discourse, as identified in Stengers (1985).

The model is structured around five key theoretical notions.

### 5.1 Organization

The notion that a self-organizing system is constituted through the structuring of relationships among components, producing an integrated whole of interacting parts.

### 5.2 Emergence

The notion that self-organizing systems exhibit novel qualities or properties at the system level, irreducible to the properties of individual components. Accordingly, such systems display at least two qualitatively distinct organizational levels: (a) the level of the parts as isolated elements, and (b) the level of the whole as the organized concatenation of those parts. In line with this notion, higher-order levels impose organizational constraints that shape the behavior of their components.

### 5.3 Autonomy

The notion that a self-organizing system exhibits a degree of independence from its environment, broadly expressed as the emergent capacity to self-determine its own dynamics and structure, as well as to respond to environmental events through endogenous self-regulation.

### 5.4 Co-evolution

The notion that a self-organizing system and its environment engage in a symmetric dynamic of reciprocal perturbations, within which each adjusts its dynamics through self-regulation, leading to coupled behaviors — that is, the emergence, on both the side of the self-organizing system and the side of its environment, of reciprocally compatible self-determined patterns of activity.

### 5.5 Closure

The notion that a self-organizing system is defined by a closed network of relations among components, which underlies the system’s emergent properties – particularly its autonomy and co-evolutionary dynamics. Based on this notion, the self-organizing system is understood as an integrative unit formed by reticular connections among elementary operations, potentially open to the development of higher-level reticular connections, via co-evolutionary dynamics with other self-organizing systems, that allow it to participate in increasingly complex self-organizational units.

According to Damiano (2009), Damiano (2012), this model aligns with the autonomous system model proposed by Francisco Varela in *Principles of Biological Autonomy* (1979/2025) based on extensive, in-depth studies on scientific research concerning natural self-organization. In that work, Varela addressed the challenge of developing a theory capable of treating the heterogeneous plurality of autonomous systems (e.g., families, ecosystems, managerial complexes, nations, clubs) in a unified manner. Specifically, Varela focused on identifying and explaining the organizational features that render systems autonomous, independently of their specific domain–whether biological, social, technical, or ecological. Varela’s solution was to propose a notion of closure that generalizes the Piagetian one (Piaget, 1967; Ceruti, 1989; Damiano, 2012), capturing the circular interdependence of components that defines autonomous or self-organizing systems. This allowed him to articulate a general framework for describing such systems across multiple levels of scientific inquiry.

As Varela writes:

“Autonomous systems are organizationally closed, that is, their organization is characterized by processes such that (1) the processes are related into a network, so that they depend recursively on each other in the generation and implementation of the processes themselves; and (2) constitute the system as a recognizable unit in the space (domain) in which the process exists. [.] The processes that specify a closed organization can be of any type and take place in any space defined by the properties of the components that constitute the process.” (Varela, 1979, p. 55)

This notion is the core of the generic model of the autonomous or self-organizing system that we adopt here. In what follows, we apply this model to the system that has formed itself around AIBO. Our ambition is to do so in line with Varela’s 1979 descriptive approach: extending the exploration of autonomy beyond the biological domain and addressing the description of autonomous systems by focusing on their organizational features rather than on their material substrate. This modeling perspective highlights the organizational interconnections at the core of the sustainability approach exemplified by the AIBO case: the interdependence of affective, technical, and social dynamics grounding sustainability-oriented human–robot ecosystems.

Having established the generic model of autonomous self-organizing systems, we now apply its organizational descriptors to examine the dynamics of the AIBO hybrid community.

## 6 Applying the model: AIBOs socio-technical ecosystems

This section provides a detailed analysis of how each core concept of the generic model of self-organizing systems can be applied to the socio-technical ecosystem surrounding AIBO.

To clarify how the theoretical framework operates in practice, in Table 1 we summarize the mapping of each key notion of the generic self-organizing system model onto the empirical dynamics of the AIBO community.

**TABLE 1:** Mapping of key self-organization concepts onto the AIBO hybrid ecosystem.

Theoretical Notion	Definition	Manifestation in the AIBO System
Organization	Structuring relationships among components to form an integrated system	The coordinated interactions between AIBO units, human owners, and A-Fun create a functional, interdependent network that maintains system coherence
Emergence	Appearance of novel properties at the system level, irreducible to the individual parts	Community-driven practices such as part salvaging, repair networks, and memorial rituals generate social and cultural phenomena beyond the technical maintenance of robots
Autonomy	System’s capacity to self- regulate and maintain coherence independently of external control.	After Sony’s withdrawal, the community organizes itself to continue AIBO lifecycles without corporate oversight, relying on local initiatives and shared knowledge
Co-evolution	Dynamic, reciprocal adaptation between the autonomous system and its external environment	The AIBO ecosystem interacts continuously with broader social, technological, and cultural contexts: Adapting to shifts in market conditions, public perceptions of robots, and available resources, while also influencing societal understandings of robotic companionship and sustainability
Closure	Internal network of operations that sustain the system’s identity and stability over time.	The closed loop of emotional attachment, technical support, part reuse, and shared rituals maintain the identity and resilience of the AIBO community over the long term

Building on the overview provided in Table 1, we now examine the specific components and relationships that constitute the AIBO socio-technical ecosystem.

The core elements of this system — the robots, their human companions, and specialized repair services — are engaged in a network of mutual dependencies that exemplifies the organizational interconnections central to the generic model of self-organizing systems.

AIBO relies on its human owner for two main reasons: first, to ensure access to electricity and routine maintenance; second, to receive the social interactions required for the optimal performance of the AIBOLife software, in models equipped to run it. In parallel, AIBO depends on technical service providers for extraordinary maintenance and component replacement, tasks that require the expertise of trained technicians.

Human users, on the other hand, develop strong emotional bonds with their robots – in some cases enhanced by the presence of AIBOLife software – leading them to prefer repairing their existing AIBO units rather than replacing them with newer models. Consequently, users come to rely on technical support companies for maintenance services and access to spare parts, no longer easily found on the market. In the specific case of A-Fun, this dependency is further deepened by the company’s additional service of conducting mortuary rites for AIBO units, a gesture aimed at honoring the users’ experiences of grief. Such practices highlight how affective and organizational dimensions intertwine, reinforcing the systemic interdependencies that sustain the community.

Furthermore, A-Fun itself is situated within a relationship of double dependency. The company relies on AIBO robots, donated by users whose units have reached the end of their operational lives, as a critical source of spare parts; at the same time, it depends on the user base to sustain demand for its services.

Taken together, these dynamics illustrate how the AIBO community aligns with the theoretical notions of the generic model of self-organizing systems, elaborated in the subsections that follow.

### 6.1 Organization

As previously introduced, the concept of organization refers to the structural relationship of elements that constitute an integrated system. Within the AIBO socio-technical ecosystem, this structure is evident in the interdependence among three primary types of components: the robotic agents (AIBO units), human users (owners), and technical support providers (e.g., A-Fun). Each of these elements possesses distinct properties that enable mutual interaction and integration within the overall system. Their interdependent relationship forms the organizational foundation of the ecosystem, allowing it to preserve its coherence and continuity over time.

It is possible to provide a more thorough description of these interdependent relationships as follows; AIBO robots depend on users for basic operational maintenance—such as power connection—and, in the case of more advanced models, require sustained human interaction to fully activate their personality-developing software (AIBOlife). They also rely on technical specialists for repair and parts replacement. Human users, in turn, develop emotional dependencies on their AIBO units, forming affective bonds that motivate continued care and resistance to disposal. This relationship is mediated by support providers, who not only maintain functionality but also organize mourning rituals for decommissioned units—thereby validating the user’s emotional investment. Lastly, technical support providers such as A-Fun depend simultaneously on human users and robotic units. AIBO users are particularly significant, as they constitute the foundation of market demand and thereby ensure the company’s continued existence. At the same time, the company relies on decommissioned AIBO robots as a vital source of replacement parts, which enables it to sustain its repair and maintenance services. The removal of any one of these elements would disrupt the network of relations and compromise the system’s integrity. This interdependence ensures the organizational closure of the system: its ability to sustain itself through internal relational dynamics.

This organization can be understood as unfolding across two interrelated dimensions: technical and societal. At the technical level, organization emerges from the interactions between technological components—such as AIBO units and their spare parts—and the human participants within the ecosystem, whose skills, knowledge, and repair practices enable effective engagement with the technology. At the societal level, organization arises through the capacity of AIBO units to engage with the human social environment via their unique embodied features. The robots’ embodiment facilitates social interaction with their owners, fostering the formation of emotional bonds. These bonds are not only experienced by individual owners but are also socially recognized within the broader community, as exemplified by A-Fun’s organization of mortuary rituals for decommissioned AIBOs, reflecting Japanese cultural and social norms. In their interplay, these technical and societal dynamics exemplify organization as both materially grounded and socially constructed. It is this dual articulation—simultaneously technological and social—that allows the AIBO ecosystem to sustain itself as an integrated unit over time, even in the absence of exogenous interventions.

### 6.2 Emergence

The notion of emergence refers to the appearance of novel, system-level patterns that cannot be reduced to the properties of individual components. In the AIBO ecosystem, emergence takes the form of two collective practices that, at the network level, function as distributed properties of the system.

The first concerns repair, salvaging, and reuse, which generate technical–material circularity. This practice arises from the joint interdependencies among users’ emotional investments, the adaptive features of the robots, and the repair expertise of specialized providers. It is not attributable to any single actor, nor was it foreseen by Sony, but emerges from the organization of the network as a whole, enabling the extension of AIBO’s lifespan and the circulation of components.

The second practice concerns the ritualization of AIBO mortuary ceremonies, developed primarily in the interplay between owners and A-Fun technicians. These ceremonies, occurring between the acknowledgment of the robot’s “death” and its dismantling for parts, were not externally imposed but emerged spontaneously from affective bonds, anthropomorphic perceptions, and cultural recognition.

Both practices illustrate how sustainability in the AIBO case is generated through intertwined technical, affective, and cultural pathways. They show that repair, reuse, and ritual are mutually reinforcing dimensions of the same sustainability-oriented dynamics. Together, they emphasize the value of an organizational reading of the AIBO hybrid community, which reveals how resilience arises through multiple, intertwined pathways.

### 6.3 Autonomy

The concept of autonomy pertains to a system’s ability to determine and regulate its own internal structure independently of external directives. The AIBO ecosystem shows a high degree of autonomy through the reorganization of its technical support structure following Sony’s withdrawal. Originally, Sony provided the entire technical assistance for AIBO units. However, after discontinuing support, this role was assumed by A-Fun, an independent company founded by a former Sony employee. This transfer of function was not exogenously directed, but arose as an endogenous, community-driven response. The strong demand from AIBO users enabled the appearance of a new actor which could take over the previous role of Sony. Despite the change in service provider, the functionality and coherence of the support system remained intact, underscoring the ecosystem’s capacity for self-regulation and structural persistence. The emergence of this new support network from within the community demonstrates the system’s autonomous ability to maintain continuity over time.

In this sense, autonomy, as defined in our theoretical reference model, can be recognized as a central organizational condition for sustainability, enabling the hybrid ecosystem to preserve and adapt its functions in response to endogenous perturbations.

### 6.4 Co-evolution

Co-evolution refers to the reciprocal adaptation of a system and its environment through ongoing perturbations that give rise to compatible patterns of activity. The AIBO ecosystem exhibits co-evolution in both economic and cultural domains.

Economically, the ecosystem adapted to market changes—specifically, the cessation of Sony’s technical support. Rather than rendering the robotic pets obsolete, this shift catalyzed the formation of alternative support services. Two key conditions enabled this adaptation: (1) the emotional commitment of AIBO users, who sought to prolong the robot’s operational life, and (2) the pre-existence of A-Fun, which had expertise in servicing vintage Sony devices. Their convergence allowed for the emergence of an adaptive repair network. Similar enterprises also appeared in other regions, such as North America and Europe, further evidencing the ecosystem’s global co-evolutionary potential. Culturally, the AIBO ecosystem adapted to the sociocultural context of Japan. The mortuary rites conducted by Buddhist monk Bungen Ōi serve as an example of cultural adaptation. Rooted in traditional beliefs, these ceremonies responded to the emotional needs of AIBO owners, reinforcing the robot’s perceived agency and social presence. In turn, these rites have shaped public understanding of the human–robot bond, contributing to a broader redefinition of affective and technological relationships.

The application of the notion of co-evolution to the AIBO case emphasizes how sustainability arises not only from preserving resources, but also from the continuous mutual adaptation between technological systems, user communities, and cultural frameworks.

### 6.5 Closure

Closure in self-organizing systems theory refers to a network of internal interactions among components that collectively sustain the system’s organization. In the case of AIBO, this closure emerges from the ongoing interplay between technical practices, social relations, and cultural recognition. When users repair or repurpose their robots, they ensure the continued functionality of the machines, which in turn reinforces the emotional bonds and social attachments that motivate further repair and preservation. These bonds are not confined to individual owners but are shared and legitimized within the broader community, a dynamic exemplified by practices such as A-Fun’s funerary rituals for decommissioned AIBOs. These rituals feed back into the ecosystem by validating collective recognition of the robots’ value, thereby amplifying the motivation to sustain them both materially and emotionally.

This recursive cycle illustrates how each dimension—technical, social, and cultural—both depends on and regenerates the others. Technical maintenance keeps robots alive as social companions; emotional attachment fuels the desire to repair and preserve; cultural practices validate and amplify the significance of these bonds, reinforcing the community’s collective efforts. None of these processes can be fully understood in isolation, since their meaning and efficacy arise from their circular interdependence. It is precisely this looping dynamic that constitutes the organizational closure of the AIBO ecosystem, enabling it to endure and adapt as a sustainability-oriented human–robot system, even in the absence of corporate support.

It is important to highlight that this network of interdependent relationships took shape after Sony’s withdrawal of technical support. We interpret this event as a system-level response to loss: a spontaneous restructuring through the formation of A-Fun as a new node, assuming the role previously occupied by Sony. This reorganization led to the development of new practices aimed at maintaining the stability of the ecosystem, particularly through the extension of AIBO’s lifespan and the preservation of the bond between the robots and their human owners.

Besides, it is worth noting that similar initiatives have emerged in other regions, including North America and Europe, where companies now offer services analogous to those provided by A-Fun. The appearance of these geographically distributed initiatives can be interpreted as another manifestation of closure at a global scale, pointing to a broader self-organizing movement emerging from the worldwide AIBO community.

**FIGURE 1:**
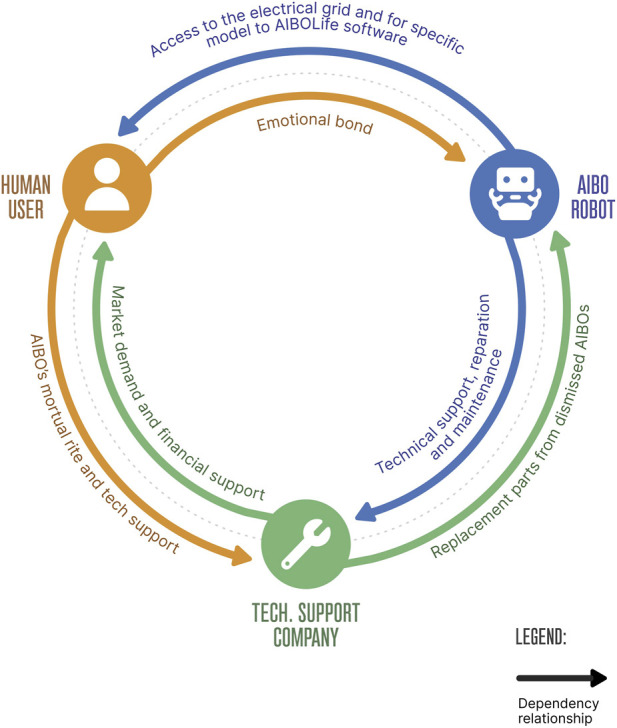
Schematic overview of the AIBO's human-robotic community as a socio-technical ecosystem. The diagram highlights the organizational interdependencies among human users, AIBO robots, and the technical support company, including emotional, economic, technical, and infrastructural forms of dependency.

To better visualize the closure and interdependencies discussed above, Figure 1 presents a schematic overview of the key relational dynamics shaping the AIBO socio-technical ecosystem.

### 6.6 A self-organizational perspective on sustainable (social) robotics

This self-organizational reading of the AIBO case opens a perspective in which sustainability in hybrid human–robot communities can be consistently framed not as a mere technical issue, but as a complex, multifactorial, and distributed process grounded in the convergence of bio-affective, cognitive, socio-cultural, and technological dimensions of human life. Such a perspective highlights the potential of self-organization theory to transform spontaneous exemplar cases into conceptual resources for developing effective approaches to the challenges of sustainable (social) robotics. The following section develops this point by examining the broader sustainability implications of this theoretical angle.

## 7 Toward a new sustainability approach for social robots

The self-organizational model we propose for the AIBO case (Table 1, Figure 1) develops the application of autonomous system modelling beyond its more classical domains — prebiotic, biological, metabiological, or technical — emphasizing how hybrid socio-technical systems can also display recognizable patterns of self-organization. This extension opens the door to a broader discussion on how such frameworks may inform sustainability strategies for social robots and, more generally, for emerging human–robot ecosystems.

In particular, the self-organizational interpretation of the AIBO community highlights a key aspect: the capacity of socio-technical systems to generate sustainability from within, in contrast to dominant models of technological consumption and rapid obsolescence. Rather than being driven by commercial incentives, the preservation of AIBO units has been sustained by affective attachments, cultural practices, and collective organizational arrangements. This virtuous dynamic demonstrates how hybrid ecosystems can foster community-driven practices that promote long-term resilience and resource conservation.

This observation suggests that sustainable practices can emerge organically from user communities themselves. For future design, this means shifting the focus beyond individual human–robot interaction or market dynamics, toward embedding repair, reuse, and stewardship protocols within the broader socio-technical systems in which robots are situated. Robots should no longer be viewed merely as consumable products destined for disposal, but as nodes within relational networks—catalysts for interaction, resources to be sustained, repaired, and reintegrated rather than discarded.

This perspective actualizes the pioneering insights of von Foerster (1960), Piaget (1967), Maturana and Varela (1980), Varela (1991) and Jantsch (1980), who first conceptualized autonomy and self-maintenance in complex systems, by applying them to hybrid socio-technical ecologies. Their work on autonomy and self-organization provides the theoretical roots for understanding how resilience can arise in artificial as well as biological domains.

From this standpoint, the robosphere acquires renewed relevance. While Colombano’s original conceptualization already acknowledged the possibility of self-repair and self-maintenance, these dimensions remained marginal. Our interpretation, grounded in the Varelian notion of organizational closure, brings sustainability to the center of the robosphere, showing how human–robot ecosystems can generate constraints and interdependencies that foster repair, reuse, and systemic stability.

Viewing the robosphere through self-organization theory frames socio-technical ecosystems as resources for enhancing sustainability, where both robots and human communities co-evolve to form relational networks oriented toward systemic self-maintenance. This approach provides the basis for a generative paradigm in robotics, where sustainability is not an add-on but a constitutive organizational principle.

Furthermore, this research trajectory may contribute to the dissemination of a culture of self-organization, supporting the development of sustainable practices within human communities. Natural self-organizing systems tend toward homeostasis through recycling, interconnectivity, and adaptation. Embedding these values into socio-technical ecologies highlights how robotics can serve as a vehicle for cultivating sustainability-oriented practices.

We thus propose that interpreting the AIBO case through the framework of self-organization is not only valuable for advancing theoretical links between self-organization and the robosphere, but also for reimagining human–robot relations in sustainability terms. This trajectory points toward a paradigm in which social robots are designed not as disposable products, but as integral nodes of sustainable, co-evolving ecosystems.

## 8 Towards principles for designing sustainable robospheric ecologies

As a first step toward envisioning sustainable socio-technical robotic ecologies, it is useful to articulate a set of preliminary principles derived from the analysis of the AIBO case through the framework of self-organization. These principles are not prescriptive rules, but orienting directions that highlight the structural, affective and social dynamics necessary for cultivating sustainable robospheric ecosystems.

### 8.1 Principle 1. Circular repairability

Robotic systems should be designed with embedded protocols that facilitate maintenance, part replacement, and the circulation of resources within the community. This requires a shift from closed, proprietary architectures toward modular and transparent designs that allow users to access, repair, and upgrade individual components without excessive technical barriers. Each component should be conceived as an independent, replaceable unit that can be removed, substituted, or repurposed without compromising the integrity of the overall system. By enabling parts to be reintroduced into the ecosystem—whether through direct reuse, recycling, or redistribution—the lifespan of robotic agents can be significantly extended. Such design strategies not only reduce waste and resource consumption but also empower user communities to take an active role in sustaining the system, reinforcing the circular dynamics that underpin organizational closure.

### 8.2 Principle 2. Networked relationality

Robotic units should be conceived not as stand-alone devices, but as nodes embedded within networks of human–robot relations. Such networks are not confined to a single dyadic interaction between a robot and its owner; rather, they encompass the broader social environment, including technicians, other users, and additional robotic agents. Each human–robot relation thus functions as one node within a wider web of interconnections, where multiple relationships intersect and reinforce one another. The existence and potential of socio-robotic communities should be explicitly considered in the design process of robotic units, with the aim of encouraging and enhancing their formation. These communities constitute valuable resources, providing opportunities for the circulation of knowledge, collaborative problem-solving, and the exchange of spare parts for repair and replacement.

### 8.3 Principle 3. Affective sustainability

The affective bond between users and robotic units must be recognized as a central driving force in human–robot relations. Such bonds often give rise to symbolic and affective practices that generate new forms of value for users, extending beyond functional utility to encompass emotional, social, and cultural dimensions. Research on social presence and artificial empathy has shown that these practices can significantly influence user behavior, motivating long-term care and preservation. Importantly, the emotional value attributed to robots is not restricted to their owners alone but can also be socially recognized by wider communities, as illustrated by rituals and cultural expressions that confer meaning on human–robot attachments. From this perspective, the design process must consider how affective practices contribute to human sociability and how they can be harnessed to promote sustainable behaviors within human–robot ecosystems.

### 8.4 Principle 4. Distributed resilience

The design of social robots should take into account the capacity of socio-technical ecosystems to reorganize themselves in response to disruption. Resilience in this context does not mean preserving an unchanged structure but rather ensuring that the system can adapt and reconfigure its organization when faced with perturbations such as the withdrawal of corporate support, the scarcity of spare parts, or the failure of technical services. Centralized dependency on manufacturers creates fragility: once production or official support ceases, the system risks collapse. The principle of distributed resilience addresses this vulnerability by encouraging design architectures that can be sustained across multiple nodes of the socio-technical ecosystem. In practice, this may involve modular hardware that can be repaired or substituted by third parties; open-source software that can be maintained by user communities; or technical documentation that facilitates grassroots repair initiatives. When responsibility for sustaining robots is shared across communities of users, technicians, and cultural institutions, resilience becomes a distributed property of the ecosystem, ensuring long-term sustainability beyond corporate lifecycles.

Together, these principles reflect how the core aspects of self-organization—emergence, autonomy, co-evolution, and closure—can be translated into orienting directions for the design and stewardship of sustainable human–robot ecosystems.

## 9 Conclusion

In this work, we have explored the AIBO case through the theoretical approach of self-organization, offering a conceptual analysis of how hybrid socio-technical systems can exhibit emergent, resilient, and sustainability-oriented dynamics. By applying the generic model of autonomous systems to the AIBO community, we have shown how practices of repair, reuse, and collective stewardship can arise spontaneously within human–robot ecosystems, generating a viable alternative to dominant paradigms of technological consumption and obsolescence.

While this study is primarily conceptual, it provides a framework that can guide future empirical research, computational simulations, and synthetic explorations. By focusing on the organizational principles underlying hybrid ecosystems, we open the door to operationalizing these concepts and testing them in diverse contexts, including the design of next-generation robots and their socio-technical environments.

Future research could build on this conceptual framework by operationalizing the organizational principles identified in the AIBO case across other human–robot ecosystems. In particular, investigating how repair, reuse, and collective stewardship can be modeled and reinforced in different contexts may open the way toward sustainability-oriented practices that integrate environmental, social, and affective dimensions. Such research would not only inform the design of new robotic systems, but could also foster a broader cultural shift toward sustainability-oriented values in human societies. In this sense, the robosphere emerges not only as an analytical framework but also as a programmatic horizon for designing robotic futures that are resilient, responsible, and socially sustainable.

## Data Availability

The original contributions presented in the study are included in the article/supplementary material, further inquiries can be directed to the corresponding author.
